# The effects of Shilajit supplementation on fatigue-induced decreases in muscular strength and serum hydroxyproline levels

**DOI:** 10.1186/s12970-019-0270-2

**Published:** 2019-02-06

**Authors:** Joshua L. Keller, Terry J. Housh, Ethan C. Hill, Cory M. Smith, Richard J. Schmidt, Glen O. Johnson

**Affiliations:** Department of Nutrition and Health Sciences, Human Performance Laboratory, University of Nebraska – Lincoln, 110 Ruth Leverton Hall, Lincoln, NE 68583-0806 USA

**Keywords:** Shilajit, Muscular strength, Collagen, Hydroxyproline, Fatigue

## Abstract

**Background:**

Shilajit is a safe, fluvic mineral complex exudate that is common to Ayurvedic medicine and is composed of fulvic acids, dibenzo-α-pyrones, proteins, and minerals. The purpose of this study was to examine the effects of 8 weeks of Shilajit supplementation at 250 mg·d^− 1^ (low dose) and 500 mg·d^− 1^ (high dose) versus placebo on maximal voluntary isometric contraction (MVIC) strength, concentric peak torque, fatigue-induced percent decline in strength, and serum hydroxyproline (HYP).

**Methods:**

Sixty-three recreationally-active men ($\overline{X}$ ± SD: 21.2 ± 2.4 yr.; 179.8 ± 6.3 cm; 83.1 ± 12.7 kg) volunteered to participate in this study. The subjects were randomly assigned to the high dose, low dose, or placebo group (each group: *n* = 21). During pre-supplementation testing, the subjects performed 2 pretest MVICs, 2 sets of 50 maximal, bilateral, concentric isokinetic leg extensions at 180°·s^− 1^ separated by 2-min of rest, and 2 posttest MVICs. Following 8 weeks of supplementation, the subjects repeated the pre-supplementation testing procedures. In addition, the groups were dichotomized at the 50th percentile based on pre-supplementation MVIC and baseline HYP. Mixed model ANOVAs and ANCOVAs were used to statistically analyze the dependent variables for the total groups (*n* = 21 per group) as well as dichotomized groups.

**Results:**

For the upper 50th percentile group, the post-supplementation adjusted mean percent decline in MVIC was significantly less for the high dose group (8.9 ± 2.3%) than the low dose (17.0 ± 2.4%; *p =* 0.022) and placebo (16.0 ± 2.4%; *p* = 0.044) groups. There was no significant (*p* = 0.774) difference, however, between the low dose and placebo groups. In addition, for the upper 50th percentile group, the adjusted mean post-supplementation baseline HYP for the high dose group (1.5 ± 0.3 μg·mL^− 1^) was significantly less than both the low dose (2.4 ± 0.3 μg·mL^− 1^; *p* = 0.034) and placebo (2.4 ± 0.3 μg·mL^− 1^, *p* = 0.024) groups.

**Conclusions:**

The results of the present study demonstrated that 8 weeks of PrimaVie® Shilajit supplementation at 500 mg·d^− 1^ promoted the retention of maximal muscular strength following the fatiguing protocol and decreased baseline HYP. Thus, PrimaVie® Shilajit supplementation at 500 mg·d^− 1^ elicited favorable muscle and connective tissue adaptations.

## Background

Nutritional supplements that contain ingredients from traditional Ayurvedic medicine have garnered substantial interest related to muscle function and connective tissue health [1–3]. Shilajit is a rock exudate found in the sedimentary rocks of Himalayan, Altai, and other mountain ranges. It is regarded as a maharasa (super-vitalizer) in Ayurveda. It contains fulvic acids as the main components along with free and conjugated dibenzo-α-pyrones (DBPs; Urolithins) and more than 40 minerals and is included as an ingredient in a number of currently available nutritional supplements [4]. Recent studies have reported that supplementation with a purified, organic form of Shilajit (PrimaVie® Shilajit) increased adenosine triphosphate (ATP) availability via improved mitochondrial function in mice [5] and increased free testosterone, total testosterone, and dehydroepiandrosterone by 19–31% in healthy men [6], which promotes increases in lean mass and muscular strength [1, 7, 8]. Thus, Shilajit supplementation may have a beneficial effect on exercise performance by enhancing fatigue-related metabolic characteristics and, potentially, increasing muscle mass and strength.

Recently, Das et al. [1] reported that consuming 500 mg·d^− 1^ of PrimaVie® Shilajit for 8 weeks upregulated extracellular matrix (ECM)-related gene expression, which promotes collagen and connective tissue integrity. Collagen is an abundant (30% of all protein in the human body) structural protein, which is found in tissues such as bone, tendons, ligaments, and muscle. Furthermore, collagen serves as a major component of the endomysium and is involved in the transmission of force generated by skeletal muscle [9]. Collagen degradation has been examined as a possible explanation for bone [10], tendon [11], and muscle -related injuries [12]. The amino acid hydroxyproline (HYP) is commonly used as an indirect biomarker of collagen degradation and the integrity of connective tissue following high-intensity exercise [11, 13–16]. Therefore, the purpose of the current study was to examine the effects of 8 weeks of Shilajit supplementation at 250 mg·d^− 1^ (low dose) and 500 mg·d^− 1^ (high dose) versus placebo on maximal voluntary isometric contraction (MVIC) strength, concentric peak torque, fatigue-induced percent decline in strength, and serum HYP. Based on previous findings [1, 6, 16] it was hypothesized that the high dose of PrimaVie® Shilajit would improve all indicators of muscular strength and collagen degradation compared to both the low dose and placebo.

## Methods

### Study design

**Fig. 1:**
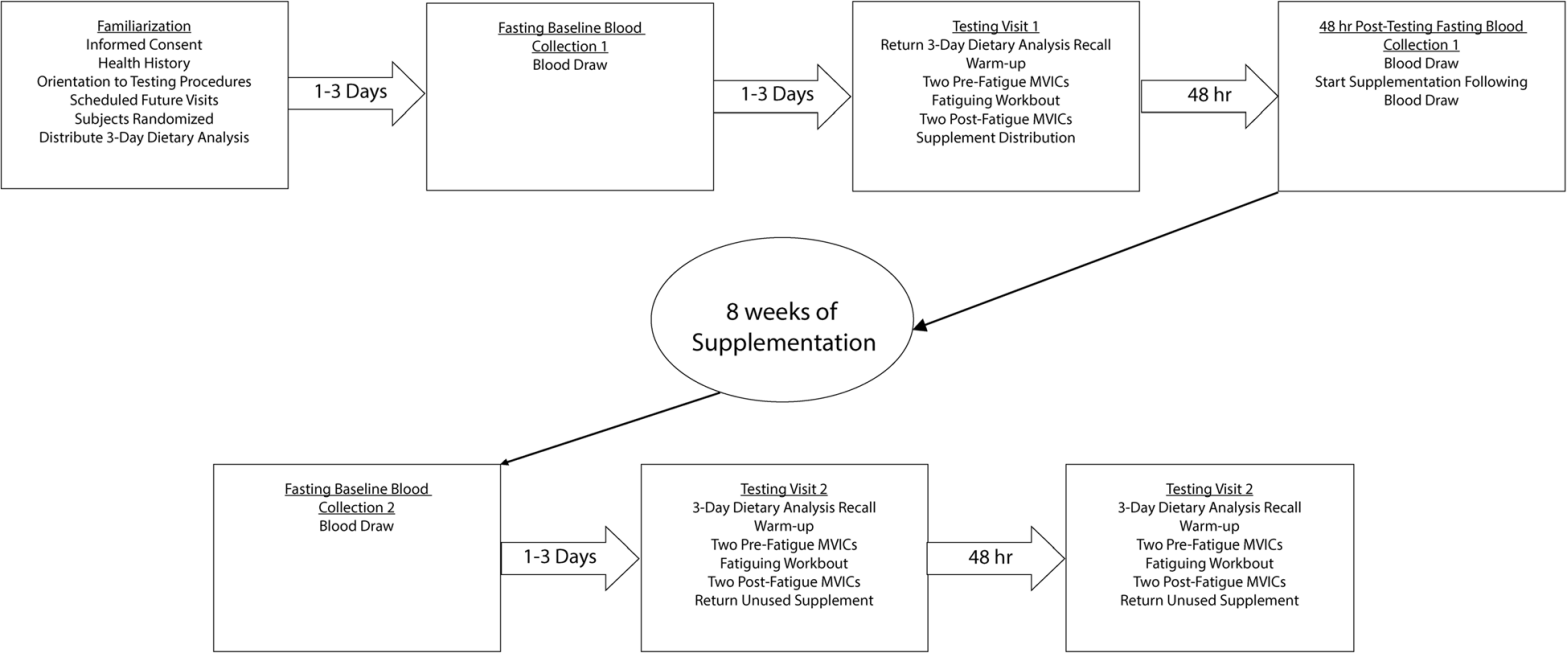
Timeline for the double-blind, placebo-controlled investigation

The present double-blind, placebo-controlled study followed the timeline in Fig. 1. The total sample (*n* = 63) was randomized into three supplement groups: low dose Shilajit (*n* = 21); high dose Shilajit (*n* = 21); and placebo (*n* = 21). The subjects visited the laboratory on seven occasions including a familiarization visit, two testing visits, and four fasting blood draws. The blood draws were used to determine serum levels of HYP as an indicator of collagen degradation. The testing visits included pre- and post-fatigue measurements of bilateral, leg extension MVIC torque values. The fatiguing protocol involved two sets of 50 maximal, bilateral, concentric, isokinetic leg extension muscle actions at 180°· s^− 1^ separated by two minutes of rest. The two testing visits were separated by eight weeks of supplementation (Fig. 1).

### Subjects

Sixty-three recreationally trained [17] men ($\overline{X}$ ± SD: 21.2 ± 2.4 yr; 179.8 ± 6.3 cm; 83.1 ± 12.7 kg) volunteered to participate in this study. The study was approved by The University of Nebraska Institutional Review Board for Human Subjects (IRB Approval #: 20171117645FB). Prior to testing all the subjects gave written Informed Consent and completed a health history questionnaire. The subjects in this study were involved in recreational sports such as rugby, basketball, softball, and flag-football and had no known cardiovascular, pulmonary, metabolic, or musculoskeletal diseases.

### Supplement and supplementation

The subjects ingested a low dose (250 mg·d^− 1^ in one capsule) of PrimaVie® Shilajit (Natreon Inc., New Brunswick, NJ), a high dose (500 mg·d^− 1^ in one capsule) of PrimaVie® Shilajit, or a placebo for eight weeks. PrimaVie® Shilajit is a purified and standardized form of Shilajit, which is composed of ≥50.0% fulvic acid as well as 10% of free DBP and DBP conjugated with chromoproteins. All capsules were identical in size and appearance.

### Procedures

#### Familiarization visit

During the familiarization visit, the subjects performed submaximal (50–70% effort) and maximal, bilateral, isometric leg extension muscle actions as well as submaximal and maximal, bilateral, concentric, isokinetic (180°· s^− 1^) leg extension muscle actions. All muscle actions were performed on a calibrated Cybex 6000 isokinetic dynamometer (Cybex International Inc. Medway, MA). Following the orientation, the subjects scheduled their first testing visit and were instructed to record their diet (MyFitnessPal, Inc.) during the three days before their scheduled testing visit 1. The subjects scheduled their first fasting (12-h overnight fast) baseline blood draw prior to their scheduled testing visit 1. The subjects were instructed to refrain from exercise for 48-h prior to testing visit 1.

#### Testing visit 1

All testing visits were scheduled before noon, and upon arrival, each subject returned their 3-day dietary recall. The subjects performed a standard warm-up of 10 bilateral, isokinetic (180°· s^− 1^) leg extension muscle actions at a self-selected intensity of approximately 50–70% effort. Following 2 min of rest, the subjects performed 2, 6 s pretest MVICs at a knee joint angle of 120° (where 180° corresponds to full leg extension at the knee). Following the determination of MVIC, the subjects completed a fatiguing protocol that consisted of 2 bouts of 50 consecutive maximal, bilateral, concentric, isokinetic leg extension muscle actions separated by 2 min of rest. Each concentric muscle action was performed through a 90° range of motion (90° to 180° of leg extension). Thus, a total of 100 maximal, bilateral, concentric, isokinetic leg extension muscle actions were completed within a 5 min period. Following the fatiguing protocol, the subjects completed 2, 6 s posttest MVICs utilizing the same procedures as the pretest MVIC trial. Following the testing visit 1 exercise procedures, the subjects completed a fasted (12 h overnight) 48-h post-exercise blood draw. In addition, the subjects were instructed not to perform additional exercise between the testing visit 1 and the 48-h post-exercise blood draw visit.

#### 48-h post-exercise blood draw visit

Upon arrival, the subjects confirmed adherence to the overnight fast as well as no additional exercise since testing visit 1. Following confirmation, a fasted blood sample was collected, and the subjects started supplementation. The subjects were then instructed to take the supplement once daily for 8 weeks (56 days) and to maintain their typical exercise and dietary habits.

#### 8 weeks Shilajit supplementation

Throughout the 8 weeks of Shilajit supplementation, the subjects were contacted biweekly to promote adherence to supplementation as well as to ensure they experienced no adverse effects possibly related to the supplement. During the last week of supplementation, the subjects scheduled the post-supplementation baseline blood draw visit as well as testing visit 2. The subjects were reminded to fast overnight prior to the post-supplementation baseline blood collection visit, to avoid performing any exercise 48-h prior to testing visit 2, and to record their diet for 3 days before testing visit 2.

#### Testing visit 2

Following the 8 weeks of Shilajit supplementation and the post-supplementation baseline blood draw visit, the subjects returned to the laboratory and provided their 3-day dietary analysis. The subjects then repeated the same testing procedures as testing visit 1. Following the completion of all testing procedures, the subjects were scheduled for their 48-h post-exercise blood collection. Again, the subjects were instructed not to exercise and to complete an overnight 12-h fast prior to the 48-h post-exercise blood draw. Furthermore, the subjects returned any remaining supplement, so that adherence could be documented.

### Determination of strength, percent decline, and collagen degradation

During testing visits 1 and 2, concentric peak torque, MVIC, percent decline in MVIC, and percent decline in peak torque were determined. For concentric peak torque, the mean of the first five repetitions of the first bout of 50 maximal, bilateral, concentric isokinetic leg extension muscle actions was defined as pretest concentric peak torque, and the mean of the last five repetitions of the second bout of 50 maximal, bilateral, concentric isokinetic leg extension muscle actions was defined as posttest concentric peak torque. In addition, pretest MVIC and posttest MVIC were defined as the greatest torque produced during the 2 MVIC muscle actions for each test. Furthermore, percent decline in MVIC and percent decline in peak torque were determined with the following equations:

$$ Percent\ Decline=\frac{Pretest\ MVIC- Posttest\ MVIC}{Pretest\ MVIC}\ast 100 $$

$$ Percent\ Decline=\frac{Pretest\ Peak\ Torque- Posttest\ Peak\ Torque}{Pretest\ Peak\ Torque}\ast 100. $$

Each subject provided 8 mL of whole blood from the antecubital vein at pre-supplementation baseline and 48 h post-exercise as well as at post-supplementation baseline and 48 h post-exercise. Thus, there was a total of 252 (4 samples per subject) blood samples. Following the blood draws, the samples were each centrifuged and stored at − 80 °C. Subsequently, 100 μL of each individual sample was aliquoted into 2 mL screw cap tubes and prepared for the tandem technique of high-performance liquid chromatography mass spectrometry. Furthermore, the column used was ACCQ-TAG Ultra C18 1.7 μm, 2.1 × 100 mm, with mobile phase A, 100% Eluent A, mobile phase B, 10:90 Eluent B: Milli-Q water, mobile phase C, Milli-Q water, and mobile phase D, 100% Eluent B. The flow rate was at 0.7 mL/min, the column oven was at 45 °C, and the runtime was 13 min per sample. Standard curves were used to calculate the concentration of HYP in the samples from the peak area detected, using the following formula:

$$ Serum\  HYP\  Concentration\ \left(\mu g\cdotp {mL}^{-1}\right)=\left( peak\ area\  HYP\right)/\left( peak\ area\ internal\ standard\right)\bullet \left( Volume\ of\ internal\ standard\bullet concentration\ of\ internal\ standard\ spike\right)/\left( slope\ from\ the\ standard\ curve\bullet \left( molecular\ weight\  HYP\right)/1000/ volume\ of\ starting\ materal\ \right) $$

### Statistical analyses

Test-retest reliability for MVIC, concentric peak torque, and baseline HYP were examined for the pre-test, pre-supplementation versus pre-test, post-supplementation measurements. Repeated measures ANOVAs were used to evaluate systematic error, and the 2,k model [18] was used to determine the intraclass correlation coefficient (ICC) and standard error of the measurements (SEM).

Each dependent variable (MVIC, concentric peak torque, and HYP levels) was statistically analyzed using a 3 (Group: Low Dose, High Dose, Placebo) × 2 (Visit: Testing Visit 1, Testing Visit 2) × 2 (Fatigue: Pretest, Posttest) mixed factorial ANOVA to examine mean differences for absolute values. In addition, ANCOVA was used to examine post-supplementation differences between groups for percent decline in MVIC, percent decline in peak torque, percent change in baseline to 48-h post-exercise HYP levels, and percent change in baseline HYP levels covarying for pre-supplementation values [19, 20]. Furthermore, paired *t-*tests were used to examine pre-supplementation versus post-supplementation for total caloric intake, carbohydrates, proteins, and fats.

Additional analyses were performed separately for sub-samples of each group that were dichotomized at the 50th percentile (median) for pre-supplementation MVIC, concentric peak torque, and baseline HYP levels. For each dependent variable, a 3 (Group: Low Dose, High Dose, Placebo) × 2 (Visit: Testing Visit 1, Testing Visit 2) × 2 (Fatigue: Pretest, Posttest) mixed factorial ANOVA was used to examine mean differences in absolute values separately for the upper 50th percentile sub-sample (*n* = 10 per group for each dependent variable) and the lower 50th percentile sub-sample (*n* = 11 per group for each dependent variable). For each sub-sample, ANCOVAs were used to examine post-supplementation differences between groups for percent decline in MVIC, percent decline in peak torque, percent change in baseline to 48 h post-exercise HYP levels, and baseline HYP levels covaried for pre-supplementation values.

Therefore, a total of nine mixed factorial ANOVAs and twelve ANCOVAs were used to examine mean differences in MVIC, concentric peak torque, percent decline in MVIC, percent decline in concentric peak torque, and HYP levels. Furthermore, significant interactions were decomposed with follow-up repeated measures ANOVAs and independent or paired samples *t*-tests. Greenhouse-Geisser corrections were applied when sphericity was not met according to Maulchy’s Test of Sphericity, and effect sizes were calculated for each comparison. Specifically, partial eta squared (${\upeta}_{\mathrm{p}}^2$) for each ANOVA and Cohen’s *d* for each Student’s *t*-test were calculated. All data are presented mean ± standard error of the mean. In addition, all statistical analyses were performed using IBM SPSS v. 25 (Armonk, NY) and an alpha of *p* < 0.05 was considered statistically significant for all comparisons.

## Results

### Test-retest reliability

Test-retest reliability was quantified using the 2,k model of Weir [18] for mean differences (systematic error), ICCs, and SEM measured pre-supplementation versus post supplementation (8 weeks apart) from the placebo group (*n* = 21). There was no significant mean difference for test versus retest for MVIC ($\overline{X}$± SD = 366.3 ± 81.8 Nm versus 346.0 ± 63.6 Nm; *p* = 0.108) or concentric peak torque (236.9 ± 56.3 Nm versus 246.1 ± 42.9 Nm; *p* = 0.415). The ICC and SEM values for MVIC and concentric peak torque were 0.75 and 52.1 Nm and 0.66 and 41.5 Nm, respectively. Test-retest reliability for HYP was determined from the baseline HYP values measured pre-supplementation versus post-supplementation (8 weeks apart) for the placebo group (*n* = 21). There was no significant mean difference for test versus retest for baseline HYP ($\overline{X}$± SD = 2.1 ± 0.90 μg·mL^− 1^ versus 2.1 ± 0.85 μg·mL^− 1^; *p* = 0.622) the ICC = 0.58, and the SEM = 0.81 μg·mL^− 1^.

### Adverse events, dietary analyses, and subject adherence

There were no reported adverse events during the course of this study. In addition, there were no significant (*p* > 0.05) differences for the dietary analyses of total caloric, carbohydrate, protein, or fat intake for pre-supplementation versus post-supplementation or between groups. Based on the number of capsules taken over the 8-week supplementation period, subject adherence was 95% (53 out of 56 capsules), 95% (53 out of 56 capsules), and 96% (54 out of 56 capsules) for the low dose, high dose, and placebo groups, respectively.

### Absolute MVIC values and percent decline in MVIC

#### Total Group (*n* = 63; 21 per group)

Table 1 provides the $\overline{X}$±SD values for the total group and subgroups for each dependent variable in the present study, There was only a significant (*p* = 0.005, ${\upeta}_{\mathrm{p}}^2$=0.122) main effect for Fatigue (collapsed across Group and Visit), and thepairwise comparison indicated that Pretest MVIC (341.8 ± 8.3 Nm) was significantly (*p* = 0.005; *d =* 0.27) greater than Posttest MVIC (323.8 ± 8.3 Nm).

**Table 1:** Descriptive Characteristics ($\overline{X}$±SD)

	Pre-Supplementation			Post-Supplementation		
	Total	Upper	Lower	Total	Upper	Lower
MVIC (Nm)						
Low Dose	376.1 ± 79.1	447.3 ± 45.8	311.5 ± 30.9	360.6 ± 95.3	431.9 ± 54.2	295.8 ± 76.3
High Dose	366.0 ± 53.5	409.8 ± 44.2	326.3 ± 18.0	335.3 ± 53.0	351.6 ± 59.7	320.5 ± 43.7
Placebo	366.3 ± 81.8	431.7 ± 60.5	306.8 ± 43.8	346.8 ± 63.6	370.4 ± 50.8	325.3 ± 68.5
% Decline MVIC						
Low Dose	16.5 ± 9.5	19.8 ± 9.1	14.1 ± 9.5	16.0 ± 12.2	18.2 ± 9.8	14.0 ± 14.1
High Dose	14.1 ± 9.4	16.6 ± 10.8	11.8 ± 7.6	9.0 ± 6.9	9.0 ± 7.5	9.1 ± 6.6
Placebo	13.0 ± 10.1	12.4 ± 10.0	14.2 ± 11.1	14.2 ± 6.9	14.8 ± 6.2	13.8 ± 7.8
Peak Torque (Nm)						
Low Dose	224.5 ± 49.0	267.4 ± 24.0	185.6 ± 28.0	253.0 ± 48.8	274.2 ± 28.6	233.7 ± 56.3
High Dose	231.8 ± 42.5	266.3 ± 20.3	200.5 ± 31.1	240.2 ± 43.4	264.0 ± 31.2	218.7 ± 42.7
Placebo	236.9 ± 56.3	283.3 ± 36.4	194.8 ± 32.1	246.1 ± 42.9	254.5 ± 43.2	238.5 ± 43.2
% Decline Peak Torque						
Low Dose	44.6 ± 15.0	46.9 ± 16.6	42.5 ± 13.8	51.5 ± 15.5	49.3 ± 15.7	53.4 ± 15.9
High Dose	42.0 ± 16.8	41.2 ± 19.4	42.7 ± 15.0	46.5 ± 16.5	50.0 ± 13.2	43.4 ± 19.2
Placebo	38.1 ± 18.9	42.2 ± 19.6	34.4 ± 18.3	41.2 ± 16.4	50.6 ± 15.6	39.7 ± 18.1
HYP (μg·mL^− 1^)						
Low Dose	2.25 ± 1.1	3.11 ± 0.9	1.48 ± 0.3	1.89 ± 0.8	2.38 ± 0.9	1.45 ± 0.5
High Dose	2.26 ± 0.8	2.87 ± 0.9	1.70 ± 0.1	1.66 ± 0.7	1.50 ± 0.6	1.81 ± 0.8
Placebo	2.12 ± 0.9	2.80 ± 0.8	1.50 ± 0.3	2.02 ± 0.9	2.40 ± 0.9	1.68 ± 0.6
% Change HYP						
Low Dose	9.63 ± 27.8	11.5 ± 34.0	7.2 ± 23.3	20.3 ± 70.2	5.5 ± 65.2	32.9 ± 78.9
High Dose	15.6 ± 32.9	23.6 ± 33.8	4.8 ± 31.3	24.0 ± 62.1	39.8 ± 83.1	11.2 ± 33.2
Placebo	17.2 ± 47.7	4.3 ± 35.7	41.1 ± 50.7	29.7 ± 92.0	0.81 ± 57.6	60.7 ± 115.7

The 1-way ANCOVA and pairwise comparisons indicated that the adjusted mean post-supplementation percent decline in MVIC was significantly (*p* = 0.021; *d* = 0.42) less for the high dose (9.1 ± 2.0%) than the low dose (15.7 ± 2.0%).

#### Upper 50th percentile (*n* = 30; 10 per group)

There was only a significant (*p* < 0.001, ${\upeta}_{\mathrm{p}}^2$=0.434) main effect for Fatigue (collapsed across Group and Visit), and the pairwise comparison indicated that Pretest MVIC (394.8 ± 9.0 Nm) was significantly (*p* < 0.001; *d* = 0.69) greater than Posttest MVIC (357.6 ± 10.5 Nm).

**Fig. 2:**
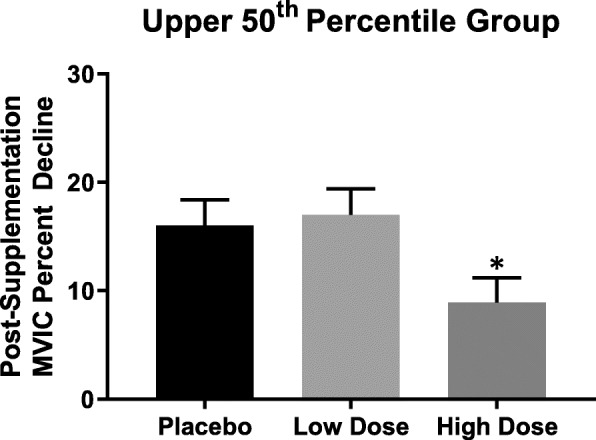
ANCOVA for the adjusted (covaried for pre-supplementation values) mean scores of post-supplementation MVIC percent decline. Note: Bars are $\overline{X}$± Standard Error of the Mean. MVIC = Maximal voluntary isometric contraction. *High dose (500 mg·d^− 1^ of PrimaVie® Shilajit) < low dose (250 mg·d^− 1^ of PrimaVie® Shilajit; *p* = 0.022) and placebo (*p =* 0.044)

The 1-way ANCOVA and pairwise comparisons indicated that the post-supplementation adjusted mean percent decline in MVIC was significantly (*p* = 0.022; *d* = 1.09 and 0.044; *d* = 0.95) less for the high dose group (8.9 ± 2.3%) than the low dose (17.0 ± 2.4%) and placebo (16.0 ± 2.4%) groups. There was no significant (*p* = 0.774) difference, however, between the low dose and placebo groups (Fig. 2).

#### Lower 50th percentile (*n* = 33; 11 per group)

There was no significant (*p* = 0.825, ${\upeta}_{\mathrm{p}}^2$= 0.013) 3-way interaction or any significant 2-way interaction (*p* = 0.992, ${\upeta}_{\mathrm{p}}^2$< 0.001; *p* = 0.494, ${\upeta}_{\mathrm{p}}^2$=0.046; *p* = 0.154, ${\upeta}_{\mathrm{p}}^2$=0.117) or significant (*p* = 0.267, ${\upeta}_{\mathrm{p}}^2$ = 0.084) main effect for Group.

The 1-way ANCOVA indicated that there was no a significant (*p* = 0.853, ${\upeta}_{\mathrm{p}}^2$ = 0.001) difference between groups for pre-supplementation or adjusted post-supplementation (*p* = 0.445; ${\upeta}_{\mathrm{p}}^2$=0.054) for percent decline in MVIC.

### Absolute concentric peak torque and percent decline in concentric peak torque

#### Total Group (*n* = 63; 21 per group)

There was no significant (*p* = 0.185, ${\upeta}_{\mathrm{p}}^2$= 0.055) 3-way or 2-way interactions containing Group (*p* = 0.582, ${\upeta}_{\mathrm{p}}^2$= 0.018; *p* = 0.524, ${\upeta}_{\mathrm{p}}^2$= 0.021) or significant (*p* = 0.572, ${\upeta}_{\mathrm{p}}^2$ = 0.018) main effect for Group.

The 1-way ANCOVA indicated there was no significant (*p* = 0.274; ${\upeta}_{\mathrm{p}}^2$=0.043) difference between groups for the adjusted (covaried for pre-supplementation values) mean post-supplementation percent decline in concentric peak torque.

#### Upper 50th percentile (*n* = 30; 10 per group)

For the low dose, there was no significant (267.4 ± 7.6 vs. 274.2 ± 9.0 Nm; *p* = 0.051, *d* = 0.26) difference between pre-fatigue, pre-supplementation, peak, concentric torque and pre-fatigue, post-supplementation, peak, concentric torque. For the high dose group, the follow-up analyses indicated that there was no significant (*p* < 0.05) 2-way interaction or main effect related to fatigue. For the placebo group, pre-fatigue, pre-supplementation, concentric, peak torque was significantly (283.3 ± 11.5 vs. 254.5 ± 13.7 Nm; *p* = 0.008, *d* = 0.72) greater than pre-fatigue, post-supplementation, peak, concentric torque.

The 1-way ANCOVA indicated that there was no significant (*p* = 0.286; ${\upeta}_{\mathrm{p}}^2$=0.092) difference between groups for the adjusted (covaried for pre-supplementation values) mean post-supplementation percent decline in concentric peak torque.

#### Lower 50th percentile (*n* = 33; 11 per group)

There was no significant (*p* = 0.349, ${\upeta}_{\mathrm{p}}^2$= 0.068) 3-way or 2-way interaction containing Group or main effect (*p* = 0.126; ${\upeta}_{\mathrm{p}}^2$=0.129) for Group.

The 1-way ANCOVA indicated that was no significant (*p* = 0.841; ${\upeta}_{\mathrm{p}}^2$=0.0012) difference between groups for the adjusted (covaried for pre-supplementation values) mean post-supplementation percent decline in concentric peak torque.

### Absolute baseline HYP and percent change in HYP

#### Total Group (*n* = 63; 21 per group)

There was no significant (*p* = 0.345, ${\upeta}_{\mathrm{p}}^2$= 0.035) 3-way or 2-way interaction as well as no main effects.

The 1-way ANCOVA indicated that there was as no significant (*p* = 0.945; ${\upeta}_{\mathrm{p}}^2$=0.002) difference between groups for the adjusted (covaried for pre-supplementation values) mean post-supplement percent decline in HYP. An additional 1-way ANCOVA indicated that there was no difference (*p* = 0.252, ${\upeta}_{\mathrm{p}}^2$ = 0.046) between groups for the adjusted (covaried for pre-supplementation values) mean post-supplementation Baseline HYP levels.

#### Upper 50th percentile (*n* = 30; 10 per group)

There was no significant (*p* = 0.256, ${\upeta}_{\mathrm{p}}^2$= 0.096) 3-way or 2-way interaction as well as no main effects.

**Fig. 3:**
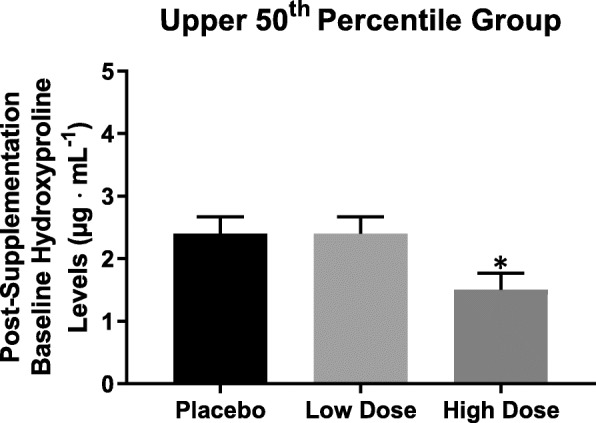
ANCOVA for the adjusted (covaried for pre-supplementation values) mean scores of post-supplementation baseline hydroxyproline. Note: Bars are $\overline{X}$± Standard Error of the Mean. *High dose (500 mg·d^− 1^ of PrimaVie® Shilajit) < low dose (250 mg·d^− 1^ of PrimaVie® Shilajit; *p* = 0.034) and placebo (*p =* 0.024)

The 1-way ANCOVA indicated that there was no significant (*p* = 0.453; ${\upeta}_{\mathrm{p}}^2$=0.022) difference between groups for the adjusted (covaried for pre-supplementation values) mean post-supplementation percent decline in HYP. An additional 1-way ANCOVA and pairwise comparisons indicated that the adjusted mean post-supplementation Baseline HYP for the high dose group (1.5 ± 0.3 μg·mL^− 1^) was significantly (*p* = 0.034; *d* = 1.00 and *p* = 0.024; *d* = 1.07) lower than both the low dose group (2.4 ± 0.3 μg·mL^− 1^) and placebo group (2.4 ± 0.3 μg·mL^− 1^) (Fig. 3).

#### Lower 50th percentile (*n* = 33; 11 per group)

There was no significant (*p* = 0.918, ${\upeta}_{\mathrm{p}}^2$= 0.006) 3-way or 2-way interaction as well as no main effect for Group. There was, however, a significant (*p* = 0.049, ${\upeta}_{\mathrm{p}}^2$= 0.124) main effect for Supplementation (collapsed across Group and Time) and the pairwise comparisons indicated that post-supplementation HYP levels (1.6 ± 0.1 μg·mL^− 1^) were significantly (*p* = 0.049, *d* = 0.75) greater than pre-supplementation (1.8 ± 0.1 μg·mL^− 1^).

The 1-way ANCOVA indicated that there was no significant (*p* = 0.760; ${\upeta}_{\mathrm{p}}^2$=0.019) difference between groups for the adjusted (covaried for pre-supplementation values) mean post-supplementation percent decline in HYP. An additional 1-way ANCOVA indicated that there was no (*p* = 0.597, ${\upeta}_{\mathrm{p}}^2$ = 0.010) difference between groups for pre-supplementation Baseline HYP levels as well as no difference (*p* = 0.540, ${\upeta}_{\mathrm{p}}^2$ = 0.042) between groups for the adjusted (covaried for pre-supplementation values) mean post-supplementation Baseline HYP levels.

## Discussion

The present study indicated that consuming 500 mg · d^− 1^ of PrimaVie® Shilajit for 8 weeks had favorable effects on the retention of maximal muscular strength following a fatiguing task. Specifically, for the total group (*n* = 63; 21 per group), the percent decline in MVIC following the fatiguing protocol was less for the 500 mg · d^− 1^ of PrimaVie® Shilajit group than the 250 mg · d^− 1^ group. In addition, the examination of the sub-sample of subjects in the upper 50th percentile of MVIC indicated that the high dose group was more resistant to fatigue and retained a greater level of maximal muscular strength than both the low dose and placebo groups.

The present study is the first to examine the effects of PrimaVie® Shilajit supplementation on fatigue-related performance outcomes using a resistance exercise model. Previous studies, however, have examined the effects of PrimaVie® Shilajit supplementation on mitochondrial indices of exercise performance that may have implications for the finding of the present study. For example, Bhattacharyya et al. [5] used a forced swimming task in mice to examine the effects of PrimaVie® Shilajit supplementation on indices of mitochondrial function including post-exercise muscle ATP concentration and adenylate energy charge. Oral PrimaVie® Shilajit supplementation (30 mg · kg^− 1^ of body weight for 4 days) resulted in a significantly greater (*p* < 0.001) post-exercise ATP concentration of 0.49 ± 0.05 μmol · g^− 1^ of muscle compared to 0.25 ± 0.05 μmol · g^− 1^ for the swimming only group without PrimaVie® Shilajit supplementation [5]. Furthermore, the adenylate energy charge, which is an index of the energy status of the cell and depends on the relative concentrations of ATP, adenosine diphosphate, and adenosine monophosphate, was significantly (*p* < 0.05) greater post-exercise for the PrimaVie® Shilajit supplementation plus swimming group (0.62 ± 0.06 units) compared to the swimming only without PrimaVie® Shilajit supplementation (0.52 ± 0.04 units). Bhattacharyya et al. [5] hypothesized that the augmented mitochondrial function, improved energy status, and upregulated ATP synthesis were the result of PrimaVie® Shilajit supplementation due to “…its potent electron transfer capacity and antioxidant activity” (p.823). The enhanced mitochondrial function reported by Bhattacharyya et al. [5] may explain the improved resistance to fatigue found in the present study for the 500 mg·d^− 1^ PrimaVie® Shilajit group, since Krustrup et al. [21] demonstrated that mitochondrial respiration contributes to the resynthesis of ATP during high intensity leg extension muscle actions similar to those used during the fatiguing protocols in the present study. Furthermore, given the beneficial findings for the upper 50th percentile of MVIC group in the present study, this explanation may be particularly applicable to athletes and/or individuals involved in resistance training. Improved mitochondrial functioning may also explain the findings of a recent unpublished, pilot study of six adult humans that found improved performance on the Harvard Step Test following 15 days of supplementation with 200 mg of PrimaVie® Shilajit [4, 22].

The results of this study indicated that 8 weeks of PrimaVie® Shilajit supplementation had no effects on the pre-fatigue MVIC, pre-fatigue concentric peak torque, or body weight values. The current study, however, involved PrimaVie® Shilajit supplementation only, but did not include a structured resistance training program. It is possible that PrimaVie® Shilajit in conjunction with resistance training may enhance strength gains as well as increases in muscle mass and body weight due to its effect on circulating testosterone levels. Pandit et al. [6] reported that 500 mg · d^− 1^ PrimaVie® Shilajit for 90 days increased baseline testosterone (4.84 ± 1.54 ng · mL^− 1^), free testosterone (15.36 ± 7.17 pg · mL^− 1^), and dehydroepiandrosterone (145.09 ± 53.17 μg · dL^− 1^) by 20.45, 19.14, and 31.35%, respectively in healthy adult men. The current study, however, did not measure hormonal responses. Based on the current findings as well as those of Pandit et al. [5], future studies should examine the effects of Shilajit supplementation on hormonal and muscular performance responses.

The current findings indicated that for the sub-sample of subjects in the upper 50th percentile for pre-supplementation HYP, the 8 weeks of PrimaVie® Shilajit supplementation at 500 mg·d^− 1^ resulted in a 29% post-supplementation decrease in baseline HYP. The reduced baseline HYP suggested a reduction in collagen degradation following supplementation. Previous studies [15, 16, 23, 24] have used HYP as an indirect indicator of collagen degradation from muscle and connective tissues. Exercise-induced muscle damage and soreness [25], connective tissue disruption [26], and elevated HYP [23, 27] often result from eccentric, but not concentric, muscles actions. The lack of acute (baseline versus 48 h post-exercise) changes in HYP in the present study following the pre-supplementation and post-supplementation fatiguing work bouts were likely due to the use of concentric-only muscle actions, which typically cause little or no muscle damage [25, 28]. Thus, the present findings suggested that the post-supplementation reduction in baseline HYP was due to a decrease in collagen degradation associated with connective tissues, but not muscle, and that 8 weeks of PrimaVie® Shilajit supplementation at 500 mg·d^− 1^ supported connective tissue health associated with tendons and ligaments [29]. Recently, Das et al. [1] found that 8 weeks of PrimaVie® Shilajit supplementation at 500 mg·d^− 1^ increased mRNA expression of collagen, the major structural protein in the skeletal muscle extracellular matrix, which accounts for 6% of the weight of tendinous muscle. Thus, the present findings, in conjunction with Das et al. [1], may have implications for competitive and recreational athletes involved in high volumes of chronic exercise who exhibit elevated exercise-induced collagen degradation [30–32].

Limitations to the current study include utilizing only a healthy, uninjured, young, convenience sample of men, whose results may not be representative of other populations. In addition, the current study’s protocol only included concentric muscle actions. Perhaps, there are different patterns of responses as a result of eccentric muscle actions. Furthermore, the present study measured serum HYP at baseline and 48-h posttest. Thus, the current study was unable to differentiate between muscle and tendon collagen degradation, however, concentric muscle actions typically do not elicit muscle damage. In addition, a detailed amino acid analysis was not conducted, which would have identified potential confounding nutrient-related differences in collagen degradation. That is, despite overall protein being similar between the groups, potential differences in protein composition were not accounted for and could have affected HYP concentrations. Also, the current study did not measure the serum concentration of the main bioactive ingredients of Shilajiit, fulvic acid as well as free and conjugated dibenzo-α-pryones. Thus, future investigations should measure the bioactivity of these ingredients to better explain performance-related adaptations. In addition, future studies should examine the effects of Shilajit supplementation in patient populations (i.e. Padget’s disease, Ehlers-Danos Syndrome, Osteogenesis Imperfecta) that are characterized by impaired collagen function as well as exercise conditions that cause muscle damage such as high intensity eccentric muscle actions in healthy individuals.

## Conclusions

In summary, the results of the present study demonstrated that 8 weeks of PrimaVie® Shilajit supplementation at 500 mg·d^− 1^ promoted the retention of muscular strength following the fatiguing protocol and decreased baseline HYP in the upper 50th percentile group. These findings were particularly associated with the stronger subjects and those with the highest pre-supplementation levels of baseline HYP. Thus, 8 weeks of PrimaVie® Shilajit supplementation at 500 mg·d^− 1^ elicited favorable muscle and connective tissue adaptations. There is, however, a limited amount of available research related to Shilajit supplementation in human subjects and the current findings should be viewed within the limitations of the design of this study. Future investigations should further examine the potential ergogenic effects of Shilajit supplementation under various exercise conditions.

## Abbreviations

ATP: Adenosine triphosphate
DBP: Dibenzo-alpha-pyrones
ECM: Extracellular matrix
HYP: Hydroxyproline
ICC: Intraclass correlation coefficient
MVIC: Maximal voluntary isometric contraction
SEM: Standard error of the measurement
